# Structure-guided identification of catalytic determinants in *ZmGA20ox* enables rational CRISPR target selection for gibberellin engineering in maize

**DOI:** 10.3389/fpls.2026.1948764

**Published:** 2026-09-09

**Authors:** Uday Kumar B. V., Sowmya H. S., U. Preethi. Praba, Gajala P. S., Jessica Kaur, Shashank S., Surinder K. Sandhu, Priti Sharma, Chandrashekar Rangu, Yogesh Vikal

**Affiliations:** 1 School of Agricultural Biotechnology, Punjab Agricultural University, Ludhiana, India; 2 Division of Agricultural Bioinformatics, ICAR-Indian Agricultural Statistics Research Institute, New Delhi, India; 3 University of Agricultural Sciences, Bangalore, India; 4 Department of Plant Breeding and Genetics, Punjab Agricultural University, Ludhiana, India; 5 Star Agriseeds, Pvt. Ltd., Hyderabad, India

**Keywords:** AlphaFold, molecular docking, molecular dynamics simulation, structure-guided genome editing, ZmGA20ox3

## Abstract

Gibberellin 20-oxidase is a key enzyme controlling gibberellin biosynthesis and represents an attractive target for engineering plant architecture and drought tolerance in maize. In this study, we developed a structure-guided computational workflow to investigate functionally important residues in maize gibberellin 20-oxidase 3 (*ZmGA20ox3*), integrating AI-based structural prediction, molecular docking, molecular dynamics simulations, and *in vitro*CRISPR/Cas9 guide RNA cleavage analysis. Structural analysis revealed the conserved Fe²^+^-binding catalytic triad (His147-Asp149-His166) surrounded by an aromatic substrate-recognition pocket comprising Phe151, Trp157, and Phe163. Docking analyses showed that native gibberellin substrates and the co-substrate 2-oxoglutarate occupied the predicted catalytic cavity, while prohexadione exhibited the most favourable predicted docking score among the tested ligands. Computational mutagenesis revealed generally less favourable predicted docking scores for the catalytic-site substitutions H147A, D149A, and H166A, whereas substitutions at aromatic substrate-recognition residues produced ligand-dependent effects. Subsequent 100 ns molecular dynamics simulations revealed distinct ligand-dependent conformational behaviours, with H147A affecting the stability of the predicted catalytic environment, whereas W157A primarily influenced ligand accommodation within the substrate-binding pocket. MM/GBSA analyses further indicated that favourable predicted ligand interactions do not necessarily imply a catalytically competent active-site configuration, highlighting the importance of productive active-site organization. Finally, *in vitro* CRISPR/Cas9 cleavage assays demonstrated sequence-specific cleavage of guide RNAs targeting the *ZmGA20ox3* regions containing H147 and W157, supporting the feasibility of targeting these sites in subsequent genome-editing experiments. Collectively, this study provides structure-guided computational insights into the potential roles of key residues in *ZmGA20ox3* and establishes a predictive framework for prioritizing candidate residues for future biochemical and genome-editing validation.

## 1 Introduction

Maize (*Zea mays* L.) is one of the world’s most important cereal crops, serving as a major source of food, feed, biofuel, and industrial raw material. Continuous improvement in maize productivity is therefore essential for sustaining global food security under increasing climatic and population pressures. Among the developmental pathways regulating crop productivity, gibberellin (GA)-mediated control of plant architecture has emerged as a major determinant of agronomic performance (Hedden, 2003; Hedden and Thomas, 2012). Gibberellins are diterpenoid phytohormones involved in stem elongation, internode expansion, floral transition, seed germination, and reproductive development (Sun, 2011). In maize, excessive vegetative elongation often predisposes plants to lodging, ultimately reducing harvest index and yield stability under intensive agricultural systems. Consequently, modulation of endogenous GA biosynthesis has become an important breeding objective for optimizing plant stature and improving resource-use efficiency in cereal crops. Central to this pathway are gibberellin 20-oxidases (GA20ox), which catalyse key late-stage oxidative reactions in the GA biosynthetic pathway and function as major rate-limiting enzymes controlling the accumulation of bioactive gibberellins (Phillips et al., 1995; Hedden and Thomas, 2012). Therefore, alteration of GA20ox activity represents a promising strategy for engineering crop architecture, manipulating plant height and stress tolerance in cereals.

The significance of gibberellin pathway manipulation in crop improvement was first demonstrated during the Green Revolution, where semi-dwarf alleles dramatically enhanced cereal productivity by improving lodging resistance and fertilizer responsiveness (Hedden, 2003). In rice, the *sd1* gene encoding GA20 oxidase became the molecular basis of semi-dwarf breeding programs and revolutionized rice production worldwide (Ashikari et al., 2002; Monna et al., 2002). Similarly, Reduced height (*Rht*) loci transformed wheat improvement through alterations in gibberellin signalling pathways (Peng et al., 1999; Ueguchi-Tanaka et al., 2007). These landmark discoveries established that targeted perturbation of gibberellin homeostasis can profoundly reshape plant architecture while enhancing agronomic performance. Despite the remarkable success of semi-dwarf breeding in rice and wheat, equivalent precision-engineered alleles remain comparatively underexplored in maize. Importantly, complete disruption of GA biosynthesis frequently results in undesirable pleiotropic effects, including severe dwarfism, impaired reproductive fitness, and reduced yield potential (Winkler et al., 1997).

Among the enzymes involved in GA metabolism, *ZmGA20ox3* represents a promising candidate for targeted genome engineering owing to its predicted involvement in bioactive gibberellin biosynthesis. *ZmGA20ox3* belongs to the 2-oxoglutarate-dependent dioxygenase (2-ODD) superfamily, a large class of non-heme Fe²^+^-dependent oxidoreductases that catalyse oxidative reactions through coordinated interactions with Fe²^+^ and 2-oxoglutarate cofactors (Hedden and Thomas, 2012; Itoh et al., 2001; Martinez and Hausinger, 2015). Members of this family possess highly conserved catalytic motifs essential for substrate recognition, oxygen activation, and catalytic turnover. Structural and biochemical analyses of GA20 oxidases from different plant species have demonstrated that histidine and aspartate residues within the catalytic pocket coordinate Fe²^+^ ions required for enzymatic activity, while surrounding hydrophobic residues contribute to substrate stabilization and ligand orientation (Helliwell et al., 2001). Recent genome-wide characterization of the maize *ZmGAox* gene family further revealed extensive structural diversification, conserved catalytic signatures, and adaptive evolutionary patterns under positive selection, highlighting the biological significance and functional complexity of GA oxidase genes in maize development and stress adaptation (Qi et al., 2025).

In this context, the present study employed a structure-guided computational framework to functionally dissect the catalytic architecture of *ZmGA20ox3* and predict rational CRISPR-editable alleles associated with altered gibberellin biosynthesis. AlphaFold-derived structural modelling, active-site characterization, in silico mutagenesis, molecular docking analyses and Molecular Dynamics Simulation were integrated to identify catalytic and substrate-recognition residues governing ligand interaction within the *ZmGA20ox3* active pocket. Both catalytic-null and partial-function mutant variants were computationally evaluated using native GA substrates and a known GA biosynthesis inhibitor to investigate their effects on substrate affinity and active-site stability. By distinguishing complete catalytic disruption from tunable reductions in substrate interaction, this study establishes a predictive framework for rational allele engineering in maize. The findings provide foundational insights for precision manipulation of gibberellin metabolism and highlight the potential of structure-guided CRISPR strategies for developing elite semi-dwarf maize genotypes with optimized agronomic architecture. Such structure-guided computational pipelines could substantially accelerate precision breeding programs by enabling informed selection of target residues prior to experimental genome editing, thereby reducing empirical uncertainty and improving the efficiency of next-generation crop improvement strategies (Zhang et al., 2020).

## 2 Materials and methods

### 2.1 Retrieval and structural modelling of *ZmGA20ox3*

The *ZmGA20ox3* protein sequence was retrieved from the maize genome resources and Ensembl Plants database using the *Zea mays* gene identifier Zm00001eb145600_T002 (LOC107521947; UniProt accession A0A1D6N5E5). The corresponding protein sequence comprises 386 amino acids. The three-dimensional structure was obtained from the AlphaFold Protein Structure Database (model identifier AF-A0A1D6N5E5-F1) and used for subsequent structural analyses (Jumper et al., 2021).The predicted three-dimensional (3D) structure of the wild-type (WT) *ZmGA20ox3* protein was downloaded in PDB format and visualized using PyMOL v2.5 (Schrödinger LLC, New York, USA) before subsequent analyses. Ramachandran plot assessment was performed using the PROCHECK server (version 3.5.4) to determine the distribution of amino acid residues within favoured, additionally allowed, generously allowed, and disallowed regions (Laskowski et al., 1993). Structural non-bonded interaction quality was evaluated using the ERRAT server (SAVES v6.0) (Colovos and Yeates, 1993). Furthermore, Verify3D analysis was conducted using the Verify3D module implemented in the SAVES v6.0 server to assess the compatibility between the generated 3D structure and its corresponding amino acid sequence environment (Eisenberg et al., 1997). The confidence of the predicted *ZmGA20ox3* structure was assessed using AlphaFold-derived pLDDT and predicted aligned error (PAE) metrics (Jumper et al., 2021). pLDDT was used to evaluate per-residue structural confidence, while PAE was used to assess the confidence of relative residue positioning. Particular attention was given to H147, D149, H166, F151, W157, and F163, which were subsequently analysed by in-silico mutagenesis, molecular docking and molecular dynamics simulations.

### 2.2 Identification of catalytic pocket and functional residues

The catalytic pocket of *ZmGA20ox3* was identified through structural inspection combined with active-site prediction analyses. Conserved catalytic residues characteristic of 2-oxoglutarate-dependent dioxygenases (2-ODDs) were identified based on structural alignment and ligand-binding cavity prediction. Particular emphasis was placed on the conserved Fe²^+^-binding catalytic triad comprising and predicted to coordinate the catalytic metal ion essential for oxidative catalysis (Huang and Madan, 1999). Aromatic and hydrophobic residues were predicted to contribute to substrate stabilization, hydrophobic interactions, and ligand orientation within the catalytic channel. Active-site pocket geometry, residue accessibility, and cavity topology were further analysed using CASTp and PrankWeb servers to identify putative substrate-binding regions and catalytic interfaces (Tian et al., 2018; Jendele et al., 2019).

### 2.3 *In silico* mutagenesis of *ZmGA20ox3*

To investigate the functional significance of catalytic and substrate-recognition residues, site-directed *in silico* mutagenesis was performed on the WT *ZmGA20ox3* structure using the Mutagenesis Wizard implemented in PyMOL v2.5 (Schrödinger LLC, New York, USA). Six mutant protein models were generated by substituting the selected residues with alanine, a widely used computational strategy to minimize side-chain steric complexity while evaluating residue-specific functional contributions (Cunningham and Wells, 1989; Kortemme and Baker, 2002). The rotamer exhibiting the lowest predicted steric clash was selected for each mutation. Following mutagenesis, all mutant structures were subjected to local energy minimization using GROMACS v2023.3 to relieve steric clashes and optimize side-chain conformations prior to molecular docking analysis (Van der Spoel et al., 2005; Abraham et al., 2015).

### 2.4 Ligand preparation

Ligands used in the present study included the gibberellin intermediates GA12, GA53, and GA20, the co-substrate 2-oxoglutarate (2OG), and the gibberellin biosynthesis inhibitor prohexadione (Olszewski et al., 2002). *ZmGA20ox3*belongs to the GA20-oxidase family of 2-oxoglutarate-dependent dioxygenases, which catalyse successive oxidation reactions in gibberellin biosynthesis. GA20ox enzymes participate in two major biosynthetic branches: the non-13-hydroxylation pathway, in which GA12 is sequentially converted to GA15, GA24, and GA9, and the early-13-hydroxylation pathway, in which GA53 is sequentially converted to GA44, GA19, and GA20. Thus, GA12 and GA53 were selected as representative upstream substrates from the two biosynthetic branches, whereas GA20 was included as the downstream product of the GA53-derived branch and a physiologically relevant metabolite associated with GA20ox activity. This selection allowed comparison of ligand recognition across distinct stages of the GA20ox-associated biosynthetic pathway. The pathway reactions and ligand-selection rationale are summarized in **Supplementary Figure 7**. Ligand structures were retrieved from the PubChem database in SDF format using their corresponding compound identifiers (Kim et al., 2023). The retrieved structures were subjected to hydrogen addition, bond-order correction, geometry optimization, energy minimization, rotatable-bond assignment, and partial-charge calculation before docking. Ligand preparation was performed using Open Babel v3.1.1, AutoDockTools (ADT) v1.5.7, and Avogadro v1.2.0 (O'Boyle et al., 2011; Morris et al., 2009; Hanwell et al., 2012). Geometry optimization was performed in Avogadro using the MMFF94 force field, followed by assignment of Gasteiger partial charges and conversion to docking-compatible PDBQT format using AutoDockTools/Open Babel. The Fe²^+^ ion was retained as a structural component of the predicted catalytic pocket during docking, based on the conserved metal-binding residues of the 2-oxoglutarate-dependent dioxygenase active site.

### 2.5 Molecular docking analysis

Molecular docking analysis was performed to investigate the interaction dynamics between WT and mutant proteins with selected ligands. Docking simulations were carried out using AutoDock Vina v1.2.3 implemented through the CB-Dock2 docking platform, which integrates cavity detection and flexible ligand docking algorithms for protein–ligand interaction prediction (Trott and Olson, 2010; Liu et al., 2022). Protein receptors were prepared using AutoDockTools v1.5.7, where polar hydrogen atoms were added, Kollman charges were assigned, water molecules and non-essential heteroatoms were removed, and receptor structures were converted into PDBQT format. Docking was performed in a site-specific manner rather than blind docking. The docking grid was centred on the catalytic pocket comprising residues H147, D149, H166, W157, F163, and F151, with an approximate grid box dimension of 24 × 24 × 24 Å to ensure complete coverage of the active-site cavity. Importantly, the Fe²^+^ ion was retained within all receptor models during the docking simulations because Fe²^+^ acts as an essential catalytic cofactor in 2-oxoglutarate-dependent dioxygenases and plays a critical role in substrate oxidation and catalytic turnover (Hedden and Thomas, 2012). This explicit Fe²^+^ treatment was restricted to the docking calculations and was not carried forward as an explicitly parameterized metal species in the subsequent molecular dynamics simulations. Docking calculations were performed using the AutoDock Vina search algorithm with an exhaustiveness value of 8, nine output binding modes (num_modes = 9), and an energy range of 3 kcal mol⁻¹. CB-Dock2 automatically generated the docking search space based on cavity prediction. The highest-ranked pose with the lowest predicted binding free energy was selected for further analyses. Since docking calculations were executed through the CB-Dock2 server, the random seed was automatically generated by the server and was therefore not manually specified. Comparative docking analysis between WT and mutant proteins was subsequently used to predict the functional impact of individual amino acid substitutions on substrate recognition and catalytic-site stability.

### 2.6 Protein–ligand interaction analysis and visualization

Protein–ligand interaction analyses were performed to characterize the binding modes of docked complexes using Discovery Studio Visualizer v21.1.0, PyMOL v2.5, and the Protein–Ligand Interaction Profiler (PLIP) web server (Salentin et al., 2015). The best-ranked docking pose for each protein–ligand complex, selected based on the lowest predicted binding free energy, was used for interaction analysis. Hydrogen bonds, hydrophobic interactions, π–π stacking, π–alkyl interactions, electrostatic interactions, salt bridges, and metal coordination involving the catalytic Fe²^+^ ion were identified using PLIP and Discovery Studio Visualizer. Three-dimensional structural visualization and figure preparation were performed using PyMOL, whereas two-dimensional interaction maps were generated using Discovery Studio Visualizer. The interaction profiles obtained from these analyses were subsequently used for comparative evaluation of ligand-binding modes among the wild-type and mutant ZmGA20ox3 protein complexes.

### 2.7 Molecular dynamics simulation

Molecular dynamics (MD) simulations were performed using GROMACS v2023.3 to investigate the structural stability and dynamic behaviour of the protein–ligand complexes (Abraham et al., 2015; Hollingsworth and Dror, 2018). Six complexes were analysed, including the wild-type (WT), H147A mutant, and W157A mutant proteins in complex with ligands 2-Oxoglutarate (2O) and GA20. H147A and W157A were selected as representative mutants of two functionally distinct residue classes identified in the structural and docking analyses: H147A represents disruption of a conserved catalytic residue, whereas W157A represents alteration of a substrate-recognition residue. This selection enabled comparison of the dynamic consequences of perturbing catalytic versus substrate-positioning residues while maintaining the computational scope of the study. The initial coordinates for MD simulations were obtained from the best docking conformations. Protein topologies were generated using the OPLS-AA/L all-atom force field (Kaminski et al., 2001), whereas ligand topologies and force-field parameters were obtained using the LigParGen web server (Dodda et al., 2017). The Fe²^+^ ion was not explicitly parameterized or included as a separate metal species in the molecular dynamics topology; therefore, Fe²^+^ coordination was not explicitly modelled during the MD simulations. Each protein–ligand complex was placed in a dodecahedral simulation box and solvated using the SPC water model (Berendsen et al., 1981). Appropriate numbers of Na^+^ and Cl⁻ ions were added to neutralize the systems. Energy minimization was carried out using the steepest descent algorithm to eliminate steric clashes and unfavourable contacts. Subsequently, the systems were equilibrated under NVT (constant Number of particles, Volume, and Temperature) and NPT (constant Number of particles, Pressure, and Temperature) ensembles for 1 ns each at 300 K and 1 bar, respectively. Temperature coupling was maintained using the Nosé–Hoover thermostat (Nosé, 1984; Hoover, 1985), while pressure coupling was achieved using the Berendsen barostat (Berendsen et al., 1984). Long-range electrostatic interactions were calculated using the Particle Mesh Ewald (PME) method (Darden et al., 1993; Essmann et al., 1995), and all covalent bonds involving hydrogen atoms were constrained using the LINCS algorithm (Hess et al., 1997). Production simulations were performed for 100 ns using a 2fs integration time step. Trajectory analyses were conducted using GROMACS built-in tools. Structural stability was evaluated by calculating the root mean square deviation (RMSD), root mean square fluctuation (RMSF), and radius of gyration (Rg) throughout the simulation period. Hydrogen-bond analyses were performed using the gmx hbond module to determine the average number of hydrogen bonds, maximum hydrogen-bond count, average hydrogen-bond distance, and hydrogen-bond angle. Hydrogen-bond occupancy (residue propensity) was subsequently calculated to identify residues involved in persistent ligand interactions during the simulation. To further characterize protein–ligand interactions, short-range electrostatic (Coul-SR) and van der Waals (LJ-SR) interaction energies were calculated using the trajectory rerun approach in GROMACS. Protein and ligand energy groups were defined through custom index files, and the production trajectories were reprocessed using the mdrun -rerun protocol. Coulombic and Lennard-Jones interaction energies between the protein and ligand were extracted from the generated energy files, and the total interaction energy was calculated as the sum of these two contributions.

### 2.8 MM/GBSA binding free energy calculation

The binding free energies of the six protein–ligand complexes were estimated using the gmx_MMPBSA v1.6.3 package (Valdés-Tresanco et al., 2021). Prior to free-energy calculations, periodic boundary conditions were removed, and the trajectories were centred and fitted to eliminate translational and rotational motions. Separate receptor, ligand, and complex groups were generated using GROMACS index files. The processed trajectories and topology files were used as inputs for MM/GBSA calculations employing the Generalized Born (GB) implicit solvent model. The binding free energy (ΔG_bind) was calculated according to:


$$\Delta G_{bind}=G_{complex}-(G_{protein}+G_{ligand})$$

where G_complex, G_protein, and G_ligand represent the free energies of the protein–ligand complex, isolated protein, and isolated ligand, respectively. The total binding free energy was decomposed into its individual energetic contributions, including van der Waals (ΔVDWAALS), electrostatic (ΔEEL), polar solvation, and non-polar solvation energy terms. The resulting ΔTOTAL values were used to compare the binding affinities of ligands 2O and GA20 toward the Wild type, H147A mutant, and W157A mutant proteins following molecular dynamics simulations.

### 2.9 *In vitro* assessment of CRISPR/Cas9 guide RNA cleavage activity

To experimentally assess the cleavage activity of the CRISPR/Cas9 guide RNAs designed to target genomic regions encoding H147 and W157 of *ZmGA20ox3*, an *in vitro* CRISPR/Cas9 cleavage assay was performed using recombinant Cas9 nuclease. DNA fragments encompassing the respective target sites were PCR-amplified from maize genomic DNA using gene-specific primers. Guide RNAs (g1: AAGAGCTGCCTGCACCGCGCGG; g2: AGGACAGCCGCTCCATCATGCGG), targeting sequences surrounding the H147 and W157 codons, respectively, were synthesized by *in vitro* transcription according to the manufacturer’s protocol (Takara Cat. Nos. 631439 and 631440). Cleavage reactions (20 μL) consisted of purified PCR amplicon, recombinant *Streptococcus pyogenes* Cas9 (SpCas9) nuclease, individual guide RNA, 10× Cas9 reaction buffer, and nuclease-free water. Reaction mixtures were incubated at 37 °C for 1 h, followed by enzyme inactivation at 65 °C for 10 min, according to the manufacturer’s instructions (Takara Cat. Nos. 631438 and 631439). The cleavage products were resolved on a 2% agarose gel, stained with ethidium bromide, and visualized using a gel documentation system. The assay was used to assess sequence-specific, gRNA-directed SpCas9 cleavage of the corresponding target regions and was not intended to establish the catalytic or structural function of H147 or W157 or to confirm generation of H147A or W157A edited alleles.

## 3 Results

### 3.1 Structural features of ZmGA20ox3

**Figure 1:**
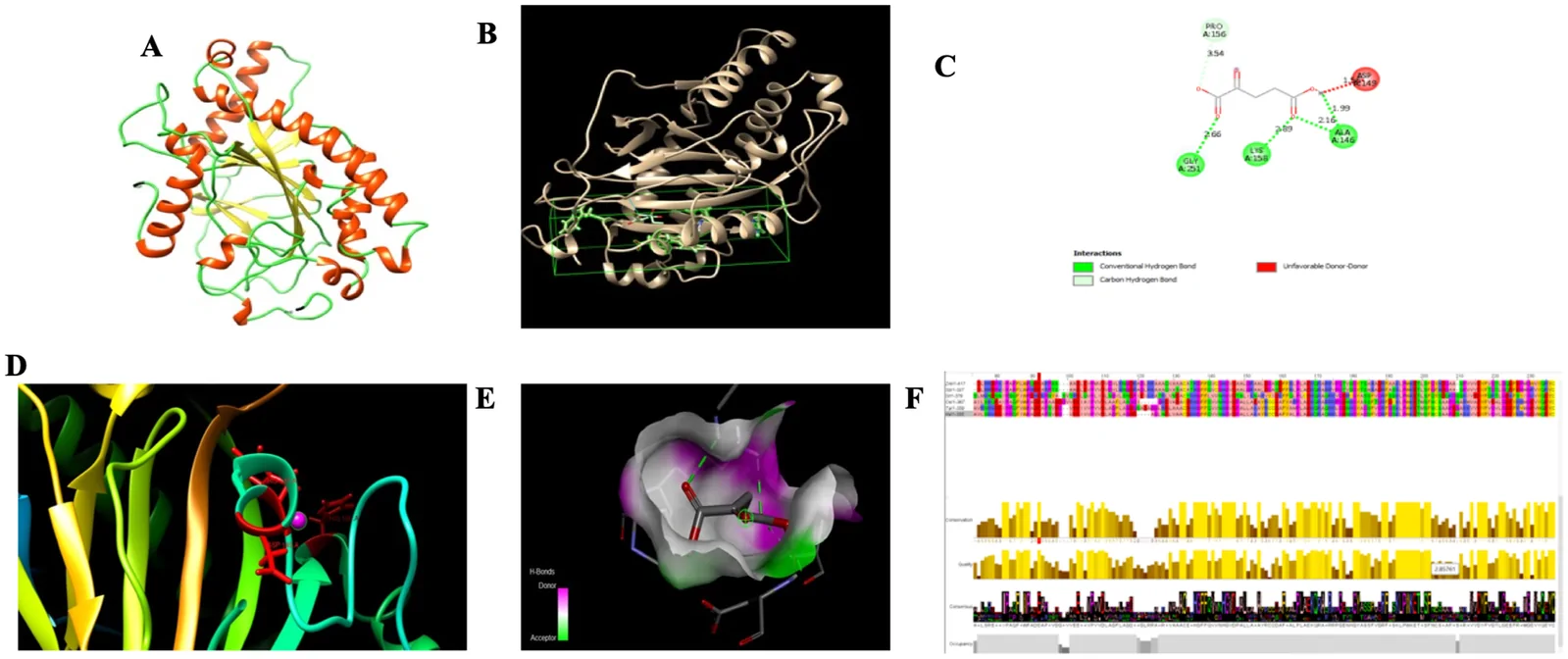
Structural characterization and catalytic-site analysis of ZmGA20ox3. **(A)** Overall three-dimensional structure of the AlphaFold-predicted ZmGA20ox3 protein showing α-helices (red), β-sheets (yellow), and loop regions (green). **(B)** Structural representation of the catalytic cavity and active-site region used for docking analysis. **(C)** Two-dimensional interaction map of the representative ligand docked within the catalytic pocket of ZmGA20ox3. **(D)** Predicted Fe²^+^-coordination geometry showing the spatial arrangement of H147, D149, and H166 relative to the modeled Fe²^+^ ion, with the corresponding coordination distances indicated. **(E)** Surface representation of ligand accommodation within the catalytic pocket of ZmGA20ox3. **(F)** Multiple sequence alignment of ZmGA20ox3 with representative GA20 oxidases from Maize with rice, wheat, sorghum and Arabidopsis showing conservation of catalytic residues within the Fe²^+^-binding motif.

The AlphaFold-predicted structure of ZmGA20ox3 exhibited a compact globular architecture comprising a conserved double-stranded β-helix (DSBH) core surrounded by α-helices and flexible loop regions, characteristic of the 2-oxoglutarate-dependent dioxygenase (2-ODD) family (**Figure 1A**). A well-defined, solvent-accessible catalytic cavity was identified within the central region of the protein, providing a structurally suitable environment for ligand binding (**Figures 1B, C**). Structural analysis identified the conserved Fe²^+^-binding catalytic triad His147–Asp149–His166, which was spatially organized to form the catalytic centre of the enzyme (**Figure 1D**). In addition, the aromatic residues Phe151, Trp157, and Phe163 were positioned adjacent to the catalytic pocket, forming a hydrophobic environment that is predicted to contribute to substrate recognition and stabilization (**Figure 1E**). Comparative structural analysis demonstrated that the catalytic motif and surrounding active-site architecture were highly conserved among plant GA20 oxidases (**Figure 1F**). Based on their structural location and predicted functional importance, His147, Asp149, His166, Phe151, Trp157, and Phe163 were selected for subsequent in silico mutagenesis, molecular docking, and molecular dynamics analyses.

### 3.2 Structural validation of the ZmGA20ox3 protein model

The stereochemical quality and structural reliability of the AlphaFold-predicted *ZmGA20ox3* model were comprehensively evaluated using PROCHECK Ramachandran analysis, ERRAT, and Verify3D. Ramachandran plot analysis showed that 246 of 279 non-glycine and non-proline residues (90.2%) were located in the most favoured regions, while 27 residues (9.7%) occurred in additionally allowed regions, 4 residues (1.4%) in generously allowed regions, and 2 residues (0.7%) in disallowed regions (**Supplementary Figures 1B, D**). The model exhibited an overall ERRAT quality factor of 89.78, indicating favourable non-bonded interaction geometry (**Supplementary Figure 1C**). Verify3D analysis showed that 91.9% of the residues achieved an averaged 3D–1D profile score ≥0.2, supporting compatibility between the predicted three-dimensional structure and its amino acid sequence (**Supplementary Figure 1A**). The AlphaFold-specific confidence metrics further supported the structural reliability of the model. The predicted *ZmGA20ox3* structure had a global mean pLDDT score of 87.44, with 73.8% of residues classified as very high confidence, 14.8% as high confidence, 2.1% as low confidence, and 9.3% as very low confidence. Importantly, the functionally investigated residues exhibited high to very high confidence: H147 (pLDDT 77.81), D149 (72.56), F151 (78.19), W157 (94.62), F163 (96.75), and H166 (93.62) (**Supplementary Figure 1E**). The predicted aligned error (PAE) values among these residues were predominantly low, ranging from 1 to 9 Å, indicating high confidence in their relative spatial arrangement within the predicted catalytic region. The mean pairwise PAE among the six functionally investigated residues was approximately 4.87 Å (**Supplementary Figure 1F**).

### 3.3 Identification of catalytic and substrate-recognition residues in ZmGA20ox3

**Figure 2:**
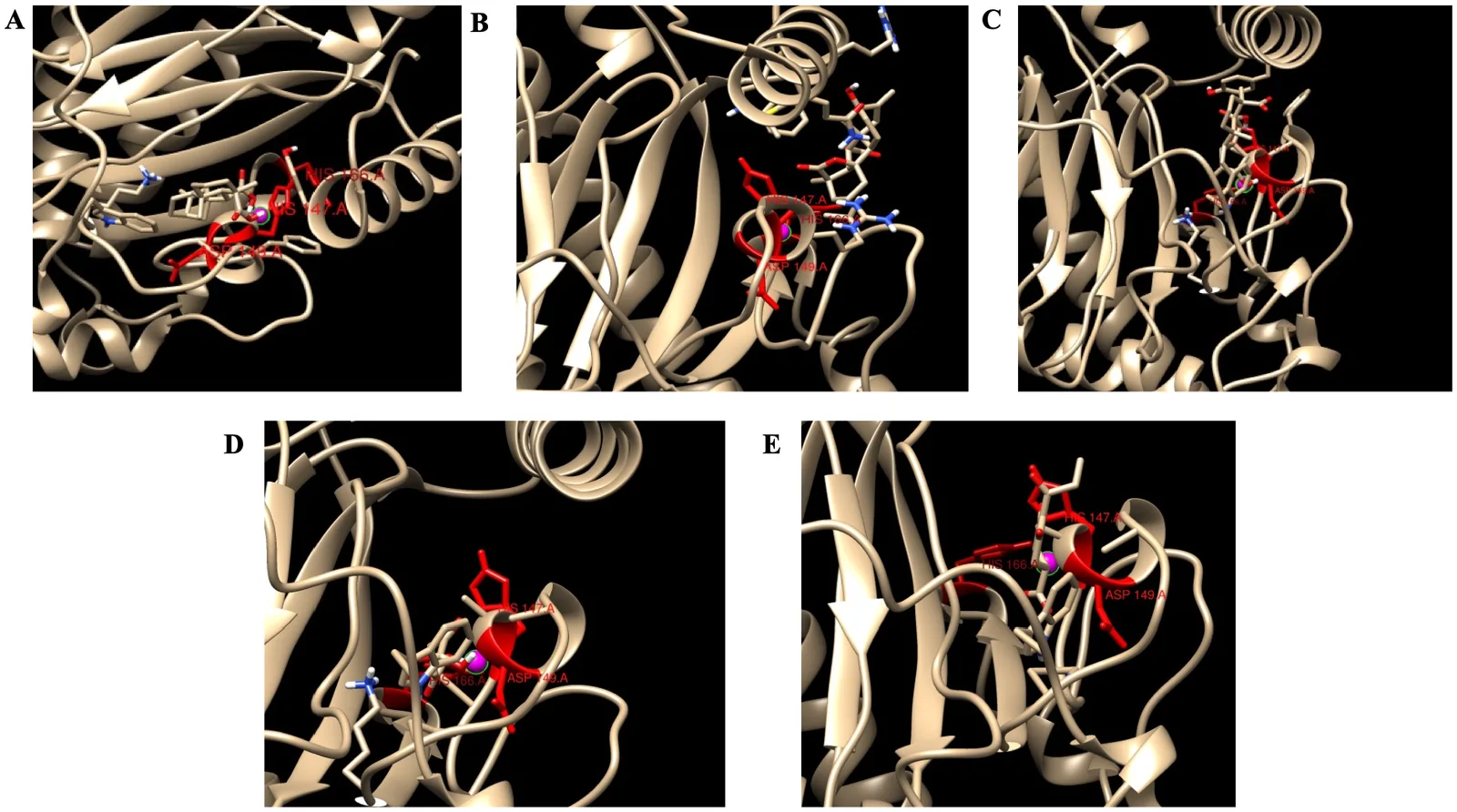
Molecular docking analysis of ligands within the catalytic pocket of wild-type ZmGA20ox3. **(A)** Docked complex of GA12 within the active-site cavity. **(B)** Binding orientation of GA20 within the catalytic pocket. **(C)** Docking pose of GA53 showing substrate accommodation within the catalytic cavity. **(D)** Interaction of the cofactor 2-oxoglutarate (2OG) with catalytic residues and Fe²^+^ coordination center. **(E)** Docking orientation of the gibberellin biosynthesis inhibitor prohexadione within the active-site pocket of ZmGA20ox3. Catalytic residues H147, D149, and H166 along with substrate-recognition residues W157, F151, and F163 are shown in stick representation surrounding the docked ligands.

Structural analysis and molecular docking identified a well-defined catalytic cavity in ZmGA20ox3 characteristic of the 2-oxoglutarate-dependent dioxygenase (2-ODD) superfamily (**Figure 2**). The catalytic pocket contained the conserved Fe²^+^-binding triad comprising His147, Asp149, and His166, which forms the catalytic core responsible for metal coordination and substrate oxidation. These residues were spatially conserved within the active-site cavity and constituted the primary catalytic framework of the enzyme. Docking of the native substrates GA12, GA20, and GA53 demonstrated that all three ligands occupied a common binding pocket adjacent to the catalytic Fe²^+^ centre (**Figures 2A–C**). The docked conformations positioned the diterpenoid backbone in close proximity to the catalytic triad, indicating a conserved substrate-binding mode. In addition to the catalytic residues, the aromatic residues Trp157, Phe151, and Phe163 formed a hydrophobic substrate-recognition pocket surrounding the ligands, suggesting roles in substrate stabilization, ligand orientation, and maintenance of the active-site architecture. Docking of the cofactor 2-oxoglutarate (2OG) confirmed its localization adjacent to the catalytic centre, preserving the canonical Fe²^+^/2-OG-dependent catalytic configuration (**Figure 2D**). Similarly, the gibberellin biosynthesis inhibitor prohexadione occupied the same catalytic cavity and overlapped with the substrate-binding channel (**Figure 2E**), indicating competitive occupation of the active site and further validating the predicted catalytic pocket. Collectively, these analyses identified His147, Asp149, and His166 as the principal catalytic residues, whereas Trp157, Phe151, and Phe163 were predicted to contribute primarily to substrate recognition and ligand stabilization. Based on their structural location and predicted functional roles, these six residues were selected for subsequent in silicomutagenesis, molecular docking, and molecular dynamics simulations to evaluate their suitability as candidate targets for structure-guided CRISPR engineering of ZmGA20ox3.

### 3.4 *In silico* mutagenesis of ZmGA20ox3

To investigate the predicted structural contributions of catalytic and substrate-interacting residues, six alanine-substituted mutant models (H147A, D149A, H166A, W157A, F151A, and F163A) were generated from the WT *ZmGA20ox3* structure (**Table 1**). Following local energy minimization, all mutant proteins retained the overall three-dimensional fold of the enzyme, indicating that the modelled single-residue substitutions did not induce major global structural perturbations. Visual inspection of the mutant structures showed that alanine substitution removed the respective side-chain functional groups while largely preserving the overall architecture of the modelled catalytic cavity. Mutations H147A, D149A, and H166A removed the side-chain groups associated with the predicted Fe²^+^-coordinating environment, suggesting that these substitutions may perturb the local catalytic-site architecture. In contrast, substitutions W157A, F151A, and F163A primarily altered the modelled hydrophobic environment surrounding the substrate-binding pocket without apparent disruption of the overall protein scaffold.

**Table 1:** *In silico*-generated *ZmGA20ox3* mutants and their predicted biological significance.

Mutant	Predicted biological rationale
H147A	Catalytic metal coordination disruption
D149A	Catalytic inactivation
H166A	Catalytic inactivation
W157A	Substrate channel alteration
F163A	Hydrophobic pocket disruption
F151A	Mild substrate orientation perturbation

### 3.5 Molecular docking analysis

Docking simulations were performed to compare the predicted binding modes and docking scores of GA_12_, GA_20_, GA_53_, 2-oxoglutarate (2OG), and prohexadione with WT and mutant *ZmGA20ox3* proteins. All ligands were accommodated within the predicted catalytic cavity containing the conserved residues H147, D149, H166, W157, F151, and F163. The Fe²^+^ ion was retained within the catalytic pocket during docking to preserve the modelled metal-containing active-site environment. Among the native gibberellins, GA_12_ showed the most favourable predicted docking score toward the WT protein, followed by GA_20_ and GA_53_, whereas prohexadione exhibited the most favourable predicted docking score among the tested ligands (**Supplementary Figure 2**). The docked poses indicated that the ligands occupied the predicted catalytic cavity with distinct orientations and residue interactions. Alanine substitutions produced residue- and ligand-dependent changes in the predicted docking scores and binding orientations. The catalytic-site substitutions H147A, D149A, and H166A showed variable changes in predicted docking scores relative to the WT protein, with the magnitude and direction of the changes depending on the ligand. Similarly, substitutions of the substrate-recognition residues W157, F151, and F163 produced variable, ligand-dependent changes in docking scores and ligand positioning. These results suggest that the investigated residues contribute differentially to the modelled ligand-binding environment of *ZmGA20ox3*; however, docking scores represent computational predictions and do not directly establish experimental binding affinity, catalytic activity, or the functional consequences of the corresponding amino acid substitutions.

### 3.6 Protein–ligand interaction analysis

**Figure 3:**
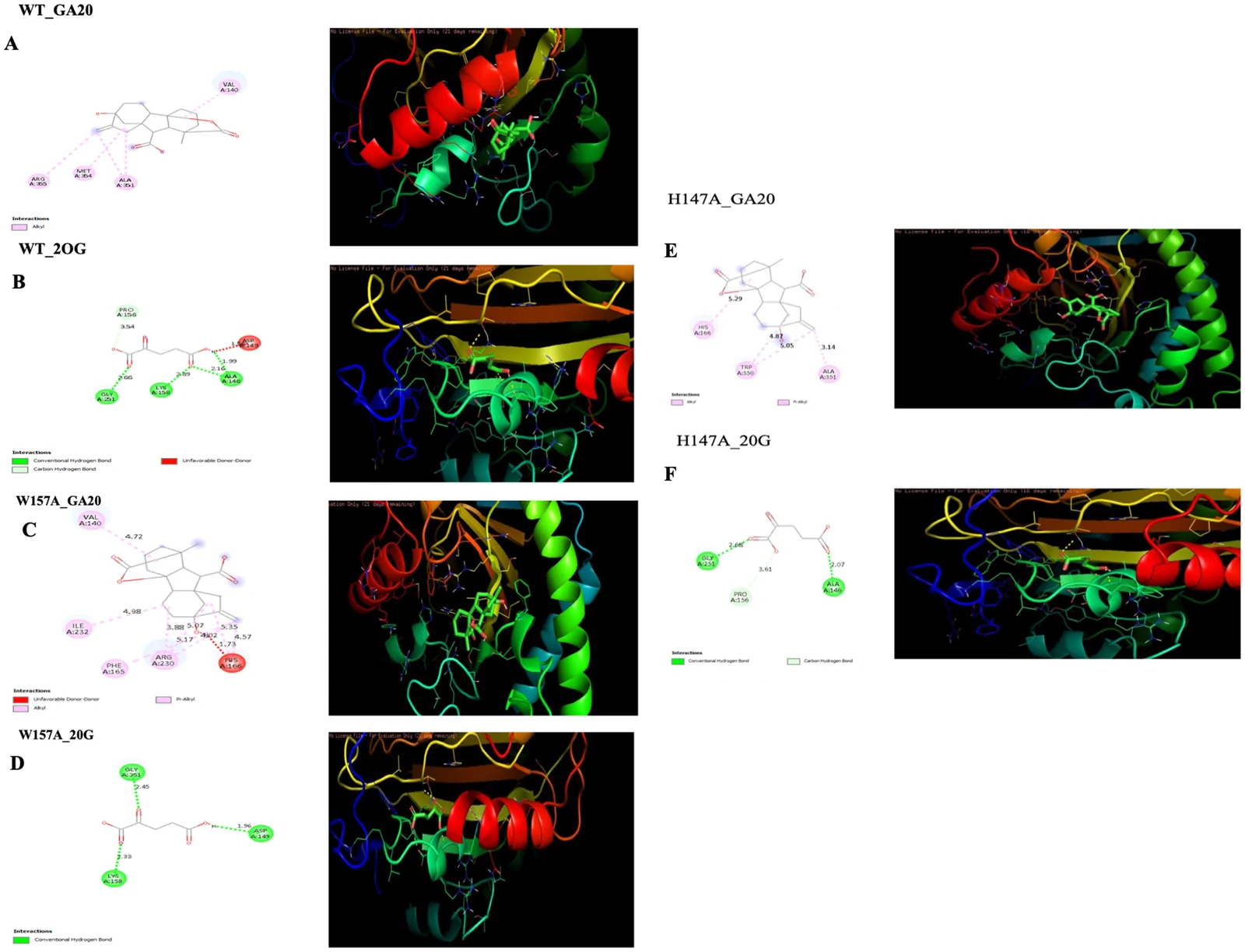
Representative two-dimensional (2D) interaction diagrams and three-dimensional (3D) binding conformations of wild-type (WT) and alanine-substituted ZmGA20ox3 proteins complexed with gibberellin A20 (GA20) and 2-oxoglutarate (2OG). **(A)** WT–GA20, **(B)** WT–2OG, **(C)** W57A–GA20, **(D)** W157A–2OG, **(E)** H147A–GA20, and **(F)** H147A–2OG. Hydrogen bonds, hydrophobic/π interactions, and ligand orientation within the catalytic cavity are shown.

Protein–ligand interaction analysis revealed that ligand stabilization within the catalytic pocket was mediated by a combination of hydrogen bonding, hydrophobic contacts, π–alkyl interactions, and coordination with the catalytic Fe²^+^ ion (**Table 2**; **Figure 3**).

**Table 2:** Protein–ligand interaction profiles of wild-type (WT) and mutant ZmGA20ox3 complexes with 2-oxoglutarate (2OG) and gibberellin A20 (GA20) obtained from molecular docking analysis.

Complex	Docking score (kcal/mol)	No. of H-bonds	Key hydrogen-bonding residues (distance, Å)	Hydrophobic/π interactions	Salt bridge/metal coordination	Important active-site residues
WT–2OG	-5.2	7	Asp149 (2.1), His166 (2.5)	W157, F151, F163	Fe²^+^	H147, D149, H166
WT–GA20	-5.3	0	Asp149 (2.3), His147 (2.8)	W157, F163	Fe²^+^	H147, D149, H166
H147A–2OG	-5.2	7	Asp149 (2.6)	F151	Fe²^+^	D149, H166
H147A–GA20	-4.2	0	His166 (2.9)	W157	None	D149
W157A–2OG	-5.2	8	Asp149 (2.4)	F151	Fe²^+^	H147, D149, H166
W157A–GA20	-6.1	1	Asp149 (2.2), His166 (2.7)	F163	Fe²^+^	H147, D149, H166

In the WT complex, GA_12_ established multiple hydrogen bonds with residues surrounding the catalytic cavity while maintaining favourable hydrophobic interactions with W157, F151, and F163. The ligand was positioned adjacent to the Fe²^+^ ion through the conserved H147–D149–H166 catalytic motif, resulting in a compact and catalytically competent binding conformation.

Substitution of catalytic residues produced pronounced alterations in interaction networks. In H147A, D149A, and H166A mutants, disruption of the catalytic coordination sphere resulted in fewer hydrogen bonds, increased ligand displacement, and reduced interaction with the catalytic metal centre. Consequently, ligand orientation within the active site became less favourable for catalysis. Conversely, mutations affecting substrate-recognition residues (W157A, F151A, and F163A) largely preserved metal coordination but weakened hydrophobic interactions responsible for substrate stabilization. Loss of aromatic side chains resulted in increased ligand mobility and altered positioning within the substrate-binding channel while maintaining partial occupancy of the catalytic pocket. Collectively, interaction profiling indicated that catalytic residues primarily govern catalytic competence through metal coordination, whereas aromatic residues contribute predominantly to substrate recognition and stabilization.

### 3.7 Molecular dynamics simulation

The structural stability and conformational dynamics of the protein–ligand complexes were evaluated through 100 ns molecular dynamics simulations using RMSD, radius of gyration (Rg), RMSF, hydrogen-bonding patterns, and protein–ligand interaction energies (Hollingsworth and Dror, 2018). The dynamic behaviour of the H147A and W157A mutant proteins was compared with that of the WT protein to assess mutation- and ligand-dependent changes in the modelled complexes.

#### 3.7.1 Root mean square deviation

**Figure 4:**
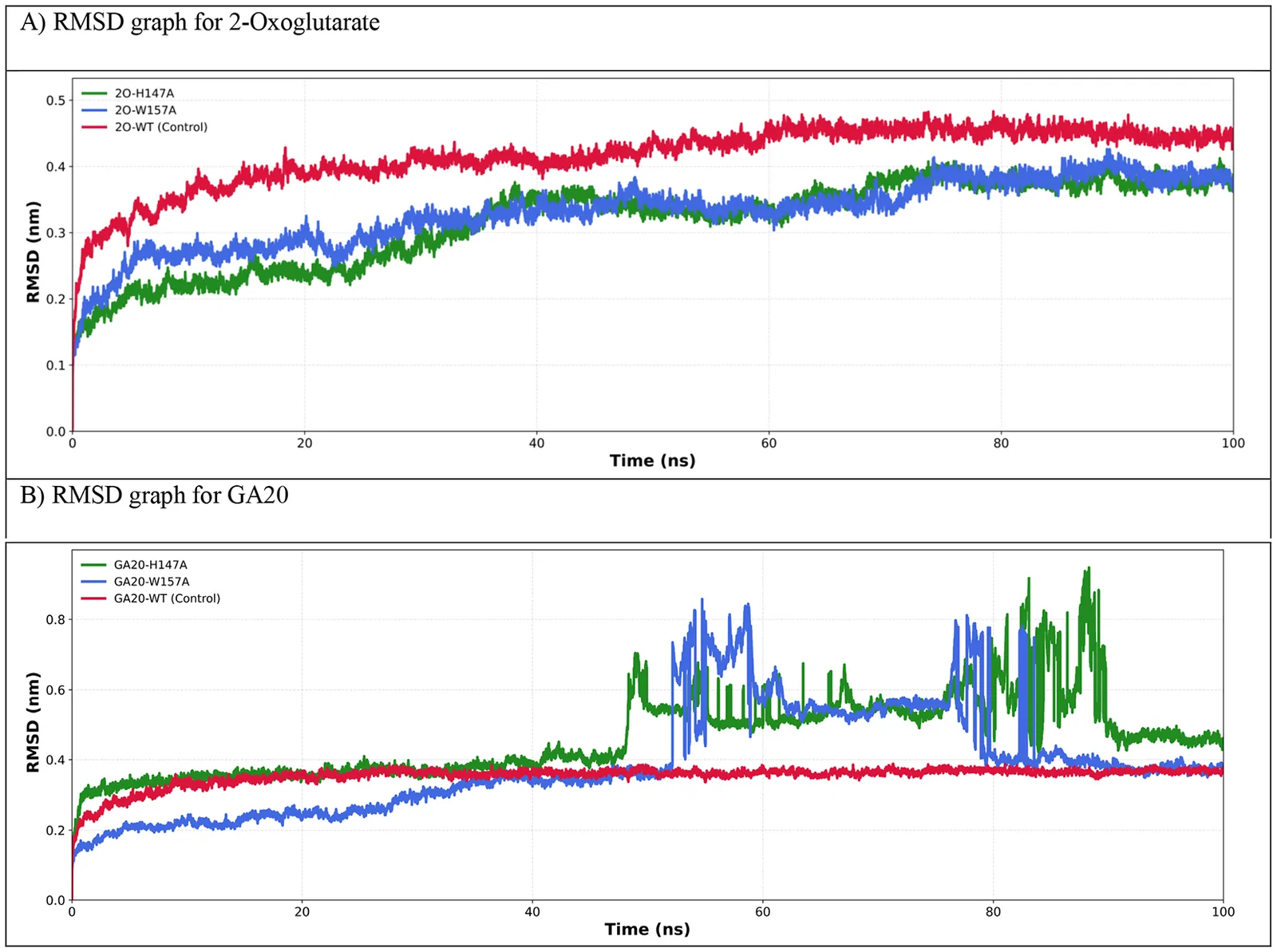
RMSD trajectories of the protein backbone and bound ligands for the WT (red), H147A mutant (green), and W157A mutant (blue) complexes with ligands **(A)** 2O and **(B)** GA20 over a 100 ns molecular dynamics simulation.

The RMSD profiles of the 2O-bound complexes showed relatively stable trajectories throughout the 100 ns simulation, with the H147A–2O and W157A–2O complexes maintaining RMSD values of approximately 0.35–0.40 nm, whereas the WT–2O complex exhibited slightly higher deviations of approximately 0.42–0.46 nm (**Figure 4A**). These differences indicate distinct conformational behaviour among the three modelled complexes, rather than providing direct evidence of enhanced ligand-binding stability in the mutants. The corresponding stability profiles were H147A > W157A > WT based on the observed RMSD values. The GA20-bound complexes exhibited more pronounced differences in their conformational trajectories (**Figure 4B**). The WT–GA20 complex remained relatively stable, with RMSD values of approximately 0.35–0.38 nm throughout the simulation. In contrast, the H147A–GA20 complex exhibited pronounced conformational changes after approximately 50 ns, reaching RMSD values close to 0.9 nm, whereas the W157A–GA20 complex showed moderate fluctuations between 50 and 80 ns before subsequently stabilizing. These observations indicate ligand-dependent differences in the conformational behaviour of the WT and mutant complexes, with the WT–GA20 complex showing the most stable trajectory among the three systems. Ligand RMSD analysis further showed the positional behaviour of 2O and GA20 within the respective binding sites during the 100 ns simulations (**Supplementary Figure 3**). The ligand RMSD profiles remained within the observed conformational range throughout the trajectories, although differences in ligand positional fluctuations were apparent among the WT and mutant complexes. These results provide additional information on ligand positional behaviour but were not used alone to infer catalytic competence or binding stability.

#### 3.7.2 Radius of gyration

**Figure 5:**
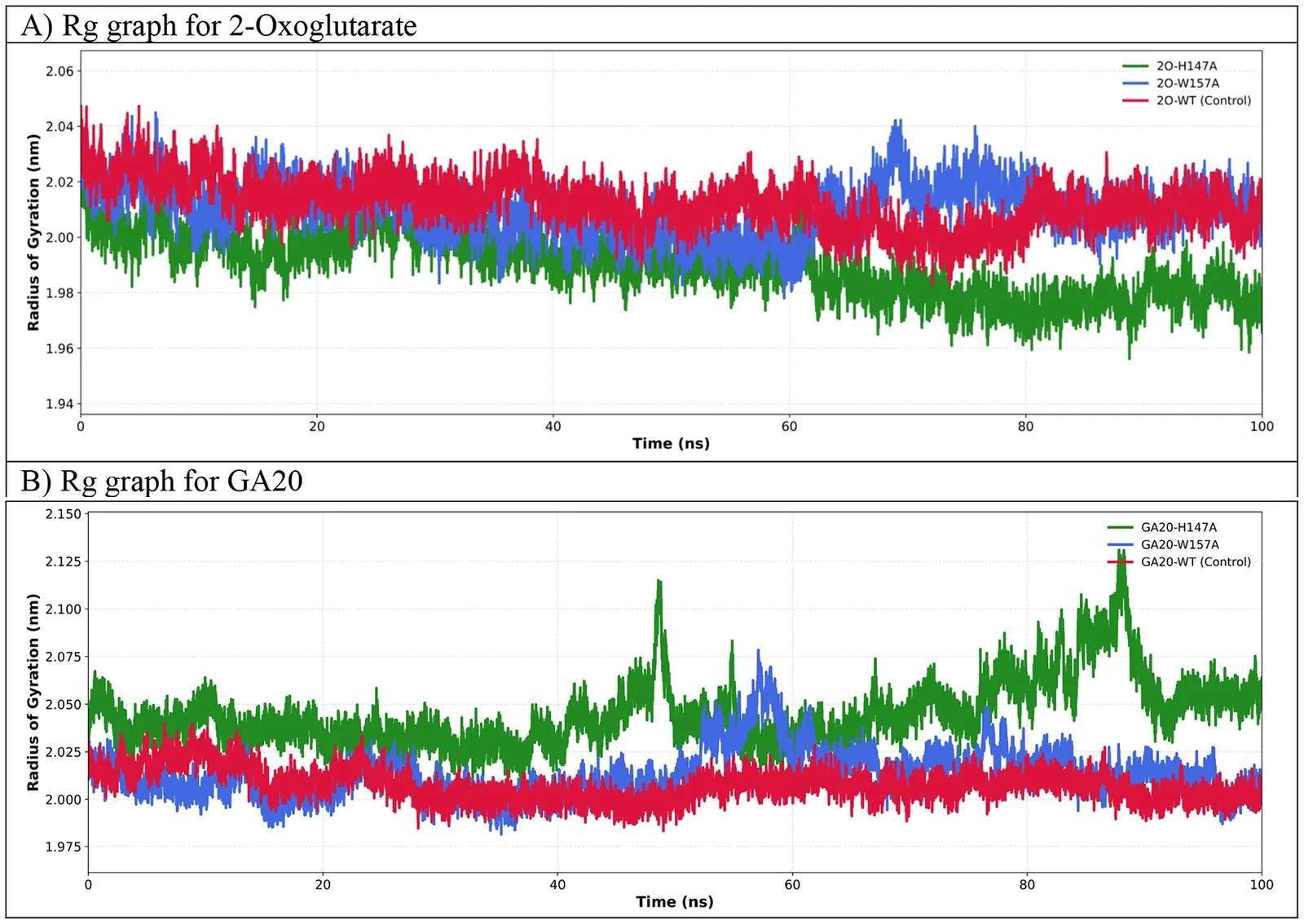
Rg profiles of the WT, H147A mutant, and W157A mutant complexes bound to ligands **(A)** 2O and **(B)** GA20 during the 100 ns molecular dynamics simulation.

The radius of gyration (Rg) was calculated to evaluate changes in the overall compactness of the protein during the 100 ns simulations (**Figure 5A**). Among the 2O-bound complexes, H147A–2O exhibited the lowest Rg values (~1.97–2.00 nm), followed by W157A–2O (~2.00–2.02 nm) and WT–2O (~2.01–2.03 nm). These profiles indicate differences in global compactness among the modelled systems, with the H147A–2O complex exhibiting the lowest calculated Rg. For the GA20-bound complexes (**Figure 5B**), WT–GA20 maintained relatively low and stable Rg values (~2.00–2.02 nm), whereas W157A–GA20 exhibited slightly higher values (~2.00–2.05 nm) and H147A–GA20 showed the highest values (~2.03–2.09 nm), with increased expansion after approximately 70 ns. These observations are consistent with the RMSD analysis and indicate greater conformational expansion of the H147A–GA20 complex during the latter part of the simulation. However, Rg values describe global structural compactness and should not alone be interpreted as direct evidence of changes in catalytic activity or experimental ligand affinity.

#### 3.7.3 Root mean square fluctuation

Residue-wise flexibility was evaluated using RMSF to identify regions exhibiting local conformational fluctuations during the 100 ns simulations (**Supplementary Figures 4A, B**). The RMSF profiles of the 2O-bound complexes were broadly similar, with higher fluctuations primarily associated with flexible surface loops and terminal regions, whereas residues surrounding the modelled ligand-binding pocket generally exhibited lower fluctuations. H147A–2O and W157A–2O showed lower average fluctuations across several regions relative to WT–2O, indicating differences in local conformational dynamics following mutation. For the GA20-bound complexes, most residues exhibited relatively low fluctuations (<0.3 nm), with larger fluctuations primarily associated with loop and terminal regions. The WT–GA20 complex showed comparatively lower overall residue fluctuations, whereas H147A–GA20 exhibited greater fluctuations in selected flexible regions and W157A–GA20 displayed an intermediate profile. The relatively stable fluctuations around the modelled binding pocket suggest that the complexes remained structurally organized during the simulations; however, these observations represent computationally predicted conformational behaviour and do not directly establish ligand retention or catalytic competence.

#### 3.7.4 Hydrogen bond analysis

The number of hydrogen bonds formed during the 100 ns MD simulation was analysed to evaluate the stability of ligand 2O within the binding pocket of the WT and mutant proteins (**Supplementary Figure 5A**). The H147A mutant exhibited the highest average number of hydrogen bonds (2.29 per frame), followed by the W157A mutant (1.51) and the WT protein (1.22), indicating a more persistent hydrogen-bonding network in the H147A complex. The H147A mutant also showed the shortest average hydrogen-bond distance (2.77 Å) together with a favourable average bond angle (13.93°), suggesting stronger hydrogen-bond interactions than the other complexes. Although the WT complex formed up to five hydrogen bonds during the simulation, these interactions were less persistent than those observed in the H147A mutant. Residue-wise hydrogen-bond occupancy further confirmed the enhanced stability of the H147A complex (**Supplementary Table 1**). ASP149 exhibited the highest occupancy (75.94%), followed by ASP364 (57.95%), CYS154 (45.61%), and SER153 (42.86%), indicating that these residues played major roles in stabilizing ligand 2O throughout the simulation. In the W157A mutant, ASP364 (54.02%) and LYS254 (33.13%) were the dominant interacting residues, whereas in the WT protein, GLU56 showed the highest occupancy (61.10%), followed by ASP364 (27.80%). Notably, ASP364 was conserved across all three complexes, suggesting that it represents a key residue for ligand recognition. Overall, the hydrogen-bond analysis indicates that the H147A mutant forms the most stable hydrogen-bonding network with ligand 2O, consistent with its lower RMSD and stronger binding interactions observed during the MD simulation. Hydrogen-bond analysis was performed to assess the stability of GA20 in complex with the WT and mutant proteins during the 100 ns simulation (**Supplementary Figure 5B**). The WT complex exhibited the highest average number of hydrogen bonds (2.26 per frame), followed by the W157A mutant (1.15), whereas the H147A mutant formed only 0.27 hydrogen bonds per frame, indicating comparatively weaker hydrogen-bonding interactions. Nevertheless, the H147A mutant showed the shortest average hydrogen-bond distance (2.86 Å), suggesting that although fewer hydrogen bonds were formed, those interactions were relatively strong. Residue occupancy analysis revealed distinct interaction patterns among the three complexes (**Supplementary Table 1**). In the WT protein, SER153 exhibited the highest occupancy (42.72%), followed by GLN358 (19.78%). The W157A mutant showed strong interactions with ARG58 (45.53%), HIS166 (31.35%), and THR60 (22.23%), whereas the H147A mutant displayed only low-occupancy interactions, with ALA147 (6.57%) representing the highest value. Overall, the WT protein maintained the most persistent hydrogen-bonding network with GA20 throughout the simulation, indicating greater binding stability than either mutant. In contrast, the H147A mutation substantially reduced hydrogen-bond formation, suggesting that this mutation adversely affects ligand binding.

#### 3.7.5 Interaction energy of complexes

**Figure 6:**
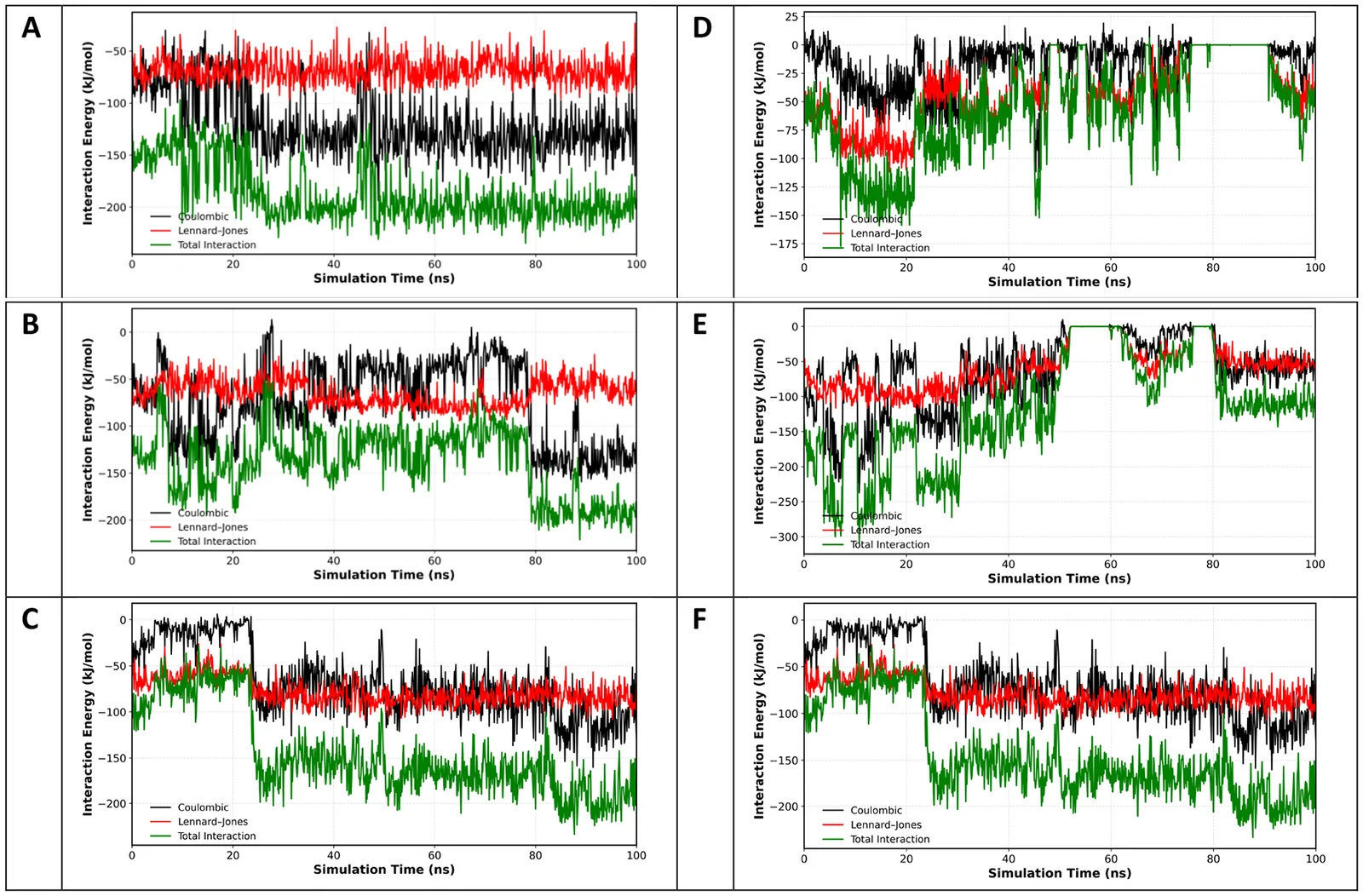
Coulombic (black), Lennard–Jones (red), and total interaction energy (green) profiles of the **(A)** H147A –2O, **(B)** W157A –2O, **(C)** WT–2O, **(D)** H147A –GA20, **(E)** W157A –GA20, and **(F)** WT–GA20 complexes during the 100 ns molecular dynamics simulation.

During the 100 ns MD simulations, short-range Coulombic (Coul-SR) and Lennard–Jones (LJ-SR) interaction energies were calculated to characterize the energetic contributions within the modelled protein–2O complexes (**Figures 6A–C**). H147A–2O exhibited the most negative total calculated interaction energy (−187.35 kJ/mol), followed by W157A–2O (−139.23 kJ/mol) and WT–2O (−108.31 kJ/mol). The H147A complex also showed more negative Coul-SR and LJ-SR contributions than the WT and W157A systems. These values indicate more favourable calculated short-range interaction-energy contributions in the H147A–2O trajectory under the applied simulation conditions, but they should not be interpreted as direct evidence that the H147A mutation increases experimental ligand affinity or catalytic activity. For GA20, the WT complex showed the most negative total calculated interaction energy (−146.40 kJ/mol), followed by W157A (−118.35 kJ/mol), whereas H147A showed the least negative value (−56.73 kJ/mol) (**Figures 6D–F**). The WT complex also displayed more favourable Coul-SR and LJ-SR contributions than H147A. Together, these results demonstrate ligand-dependent differences in the calculated energetic profiles of the WT and mutant complexes, highlighting that the effect of a residue substitution cannot be inferred from a single ligand or energetic descriptor.

#### 3.7.6 MM/PBSA binding free energy analysis for ligands 2O and GA20

The binding free energy of ligand 2O was calculated using the gmx_MMPBSA v1.6.3 tool over the 100 ns MD simulation to evaluate the energetic favourability of the protein–ligand association. As summarized in **Table 3**, the H147A mutant exhibited the most favourable calculated binding free energy (ΔTOTAL = −16.98 ± 2.58 kcal/mol; SD, standard deviation; SEM, standard error of the mean = 0.26), followed by the W157A mutant (−14.68 ± 4.07 kcal/mol; SEM = 0.41) and the WT protein (−11.42 ± 2.87 kcal/mol; SEM = 0.29). The H147A mutant also showed the most favourable van der Waals (ΔVDWAALS = −18.15 kcal/mol) and electrostatic (ΔEEL = −22.15 kcal/mol) contributions, resulting in the most favourable calculated binding free energy among the three complexes. Overall, the MM/GBSA results indicate a more energetically favourable association of 2O with the H147A mutant than with the WT and W157A proteins.

**Table 3:** MM/PBSA binding free energy components of ligands 2O and GA20 in complex with wild-type and mutant proteins.

2O			
Energy component (kcal/mol)	H147A mutant	W157A mutant	WT (wild)
ΔVDWAALS	−18.15	−16.00	−15.41
ΔEEL	−22.15	−23.36	−10.15
ΔGGAS	−40.31	−39.42	−25.56
ΔTOTAL(Binding Free Energy)	−16.98 ± 2.58; 0.26	−14.68 ± 4.07; 0.41	−11.42 ± 2.87; 0.29
GA20			
Energy component (kcal/mol)	H147A mutant	W157A mutant	WT (wild)
ΔVDWAALS	−15.87	−24.17	−17.78
ΔEEL	−5.70	−55.19	−12.98
ΔGGAS	−21.56	−79.37	−30.76
ΔTOTAL (Binding Free Energy)	-5.03 ± 2.46;0.24	-17.50 ± 4.36; 0.44	-10.21 ± 2.43; 0.24

Values are presented as mean ± SD; SEM is given in parentheses. SD and SEM represent variability across the trajectory frames sampled for the MM/GBSA calculation and do not represent variability among independent MD replicates.

Similarly, the binding free energy of GA20 was estimated using the gmx_MMPBSA v1.6.3 method throughout the 100 ns simulation. As shown in **Table 3**, the W157A mutant exhibited the most favourable calculated binding free energy (ΔTOTAL = −17.50 ± 4.36 kcal/mol; SEM = 0.44), followed by the WT protein (−10.21 ± 2.43 kcal/mol; SEM = 0.24), whereas the H147A mutant displayed the weakest calculated binding free energy (−5.03 ± 2.46 kcal/mol; SEM = 0.24). The more favourable calculated binding free energy of the W157A complex was primarily associated with its highly favourable electrostatic (ΔEEL = −55.19 kcal/mol) and van der Waals (ΔVDWAALS = −24.17 kcal/mol) contributions, resulting in the most favourable gas-phase interaction energy (ΔGGAS = −79.37 kcal/mol). Overall, the MM/GBSA analysis indicates that the W157A–GA20 complex has the most favourable calculated binding free energy, whereas the H147A–GA20 complex shows less favourable protein–ligand energetics.

### 3.8 *In vitro* validation of CRISPR/Cas9 guide RNA cleavage activity

To experimentally assess the cleavage activity of the designed CRISPR/Cas9 guide RNAs targeting genomic regions encoding H147 and W157 of *ZmGA20ox3*, an *in vitro* Cas9 cleavage assay was performed. PCR amplification generated the expected target amplicon, and incubation with recombinant SpCas9 in the presence of the respective guide RNAs resulted in cleavage products corresponding to the predicted target sites (**Supplementary Figure 6**). The control reaction lacking guide RNA did not show guide-dependent cleavage. These results demonstrate that the designed gRNAs can direct sequence-specific SpCas9 cleavage of the corresponding *ZmGA20ox3* DNA targets under the tested *in vitro* conditions. Importantly, this assay assesses gRNA-directed DNA cleavage activity and does not establish the catalytic or structural function of H147 or W157, nor does it demonstrate generation of H147A or W157A alleles in planta.

## 4 Discussion

Gibberellin 20-oxidases (GA20oxs) are key enzymes in the gibberellin biosynthetic pathway, catalysing successive oxidation reactions that regulate endogenous GA levels and consequently influence plant height, architecture, reproductive development, and environmental adaptation (Yamaguchi, 2008; Hedden and Thomas, 2012). Despite their biological importance, structural information for crop GA20 oxidases remains scarce, limiting mechanistic understanding of substrate recognition and catalysis. By integrating AlphaFold-based structural prediction, molecular docking, molecular dynamics (MD) simulations, computational mutagenesis, and experimental CRISPR/Cas9 target validation, this study provides a comprehensive structural framework for understanding ZmGA20ox3 function and demonstrates how structural information can guide rational genome-editing strategies (Zhang et al., 2020). The predicted ZmGA20ox3 structure adopted the conserved double-stranded β-helix (DSBH) fold characteristic of Fe²^+^/2-oxoglutarate-dependent dioxygenases (2-ODDs), supporting its classification as a canonical GA20 oxidase (Martínez et al., 2001). This structural scaffold forms the catalytic core responsible for coordinating Fe²^+^ and 2-oxoglutarate (2-OG), a feature that has been conserved throughout the plant 2-ODD superfamily despite considerable sequence divergence (Hausinger, 2004; Kawai et al., 2014; Lange and Hedden, 2020). Furthermore, structural validation using Ramachandran analysis, Verify3D, and ERRAT confirmed excellent stereochemical quality, indicating that the AlphaFold model provides a reliable platform for mechanistic investigations, consistent with previous evaluations of AlphaFold-predicted structures (Jumper et al., 2021; Varadi et al., 2022).

Structural analysis identified the highly conserved H147-D149-H166 catalytic triad corresponding to the canonical HXD … H motif responsible for Fe²^+^ coordination in 2-ODDs. Disruption of this motif is known to abolish oxygen activation and oxidative decarboxylation of 2-OG, resulting in catalytic inactivation (Hausinger, 2004; Clifton et al., 2006). In contrast, Phe151, Trp157, and Phe163 formed a conserved hydrophobic environment surrounding the catalytic centre. Aromatic residues commonly stabilize substrates through hydrophobic packing and π-mediated interactions while facilitating proper substrate orientation without directly participating in catalysis (Bissantz et al., 2010). Their spatial organization therefore suggests a complementary role in substrate recognition and catalytic efficiency. Docking analyses supported the functional relevance of the predicted active site. All endogenous substrates (GA_12_, GA_20_ and GA_53_), together with the co-substrate 2-OG and the growth retardant prohexadione, occupied the same catalytic cavity. GA_12_ exhibited the strongest affinity among the natural substrates, consistent with its role as an early substrate in gibberellin biosynthesis, whereas prohexadione displayed the highest overall affinity, reflecting its established role as a competitive inhibitor of GA20 oxidases (Yamaguchi, 2008; Rademacher, 2000). Protein-ligand interaction analyses further indicated that catalytic residues primarily maintained the Fe²^+^ coordination geometry, whereas the neighbouring aromatic residues stabilized ligand orientation through hydrophobic interactions. Such functional partitioning between catalytic and substrate-positioning residues is widely recognised among metalloenzymes and is essential for efficient catalysis (Hausinger, 2004; Bissantz et al., 2010). Computational alanine mutagenesis clearly distinguished these two functional classes. Mutations within the catalytic triad (H147A, D149A and H166A) markedly reduced ligand affinity and altered binding orientation, confirming the indispensable role of the HXDH motif in maintaining a catalytically competent active site. Conversely, mutations of Phe151, Trp157 and Phe163 produced comparatively modest effects on docking while preserving ligand accommodation within the catalytic pocket.

Because molecular docking provides only static snapshots of protein-ligand interactions, 100 ns molecular dynamics simulations were performed to investigate the dynamic behaviour of wild-type ZmGA20ox3 together with the H147A and W157A mutants in complex with 2-OG and GA20. All simulated complexes maintained relatively consistent overall conformational behaviour throughout the simulations, supporting their use for comparative dynamic analyses. For the 2-OG-bound complexes, H147A consistently exhibited lower RMSD and radius of gyration (Rg) values than the wild type, indicating lower conformational deviation and greater structural compactness. However, increased structural rigidity should not be interpreted as enhanced catalytic function because many enzymes require conformational flexibility to facilitate substrate binding, oxygen activation and product release (Henzler-Wildman and Kern, 2007). In contrast, the GA20-bound wild-type complex exhibited the lowest overall conformational deviation throughout the simulation, whereas H147A displayed progressively larger conformational deviations after approximately 50 ns, indicating greater conformational fluctuations relative to the wild type.W157A consistently exhibited intermediate conformational behaviour relative to the WT and H147A complexes. H147A showed reduced fluctuations in the 2-OG-bound state but increased flexibility around the catalytic centre in the presence of GA20, indicating ligand-dependent differences in local conformational dynamics. Although H147A formed persistent hydrogen bonds with 2-OG, these interactions may represent stabilisation of a non-productive binding mode rather than productive catalysis, a phenomenon frequently observed in catalytic mutants of metalloenzymes (Warshel et al., 2006). Conversely, the wild-type enzyme maintained the most persistent hydrogen-bonding network with GA20, whereas W157A retained an intermediate hydrogen-bonding profile, consistent with differences in substrate positioning. Interaction-energy and MM/GBSA analyses further supported the observed differences in protein–ligand energetics, with H147A exhibiting the most favourable interaction energies and calculated binding free energy toward 2-OG, whereas the wild-type protein displayed substantially more favourable binding energetics with GA20. Although apparently paradoxical, these findings illustrate a fundamental principle of enzymology: catalytic efficiency depends on productive substrate orientation and transition-state stabilisation rather than maximal binding affinity alone (Warshel et al., 2006). Loss of His147 likely stabilises 2-OG in a non-productive conformation, whereas substitution of Trp157 primarily affects substrate positioning while preserving much of the catalytic framework. These findings suggest that the catalytic and substrate-recognition residues contribute differently to the dynamic organisation of the active site. However, because Fe²^+^ was not explicitly parameterized in the MD simulations, direct changes in Fe²^+^ coordination could not be evaluated. Therefore, the present MD results are interpreted primarily in terms of protein conformational stability and protein–ligand dynamics. Metal-aware MD simulations incorporating an explicitly parameterized Fe²^+^ centre would be required to directly evaluate the effects of these mutations on metal coordination and catalytic geometry. Another computational limitation is that MD simulations were performed for two representative mutants, H147A and W157A, rather than all six generated alanine mutants. These mutants were selected to represent catalytic and substrate-recognition residues, respectively, enabling comparison of their distinct effects on protein–ligand dynamics. MD simulations of all six mutants, together with the apo protein, would provide a more comprehensive assessment of mutation- and ligand-dependent conformational changes. In addition, only a single 100 ns MD trajectory was generated for each system. Therefore, the observed conformational changes may reflect stochastic fluctuations and cannot definitively establish mutation-specific effects. Independent replicate simulations would be required to assess their reproducibility and statistical robustness. Collectively, the MD simulations provide supportive evidence for distinct dynamic effects of catalytic and substrate-recognition mutations, while the docking results provide structural evidence for their predicted roles in substrate recognition and accommodation.

Beyond providing structural insights into *ZmGA20ox3*, this study demonstrates the potential value of integrating structural information with genome-editing target prioritization. Current CRISPR/Cas9 target selection primarily relies on nucleotide-level parameters, including PAM availability, guide efficiency, and predicted off-target activity, while the structural and potential functional relevance of the encoded amino acids is less frequently considered (Hsu et al., 2014; Doench et al., 2016). Here, AlphaFold-based structural modelling, evolutionary conservation, computational mutagenesis, molecular docking, and molecular dynamics simulations were integrated to prioritize candidate residues according to their predicted structural and functional relevance. Catalytic residues such as His147 and substrate-recognition residues such as Trp157 were subsequently selected as candidate regions for future experimental genome-editing studies. *In vitro* CRISPR/Cas9 cleavage assays demonstrated sequence-specific cleavage of genomic regions containing His147 and Trp157, supporting the technical feasibility of targeting these regions with the selected guide RNAs. Importantly, the cleavage assay does not validate the predicted catalytic or substrate-recognition functions of these residues, demonstrate the generation of H147A or W157A edited alleles, or establish their effects on enzyme activity or plant phenotype. Rather, it provides experimental evidence that the selected guide RNAs can recognize and cleave the corresponding target DNA sequences under *in vitro* conditions. Generation and molecular characterization of genome-edited plants, together with biochemical and phenotypic analyses, will be required to experimentally test the functional consequences predicted by the computational analyses. Because transformation and regeneration remain labour-intensive and genotype-dependent in maize (Ishida et al., 2007; Lowe et al., 2016), structure-guided prioritization of candidate residues may help focus subsequent experimental validation. More broadly, the workflow presented here provides a predictive framework for prioritizing structurally and computationally supported candidate residues for future genome-editing and functional studies of plant proteins.

## 5 Conclusion

This study provides a comprehensive structural and functional characterization of maize gibberellin 20-oxidase 3 (ZmGA20ox3) through an integrated computational and experimental framework. AlphaFold-based structural modelling, molecular docking, computational mutagenesis, molecular dynamics simulations, and MM/PBSA free-energy analyses collectively demonstrated that the conserved catalytic triad (His147-Asp149-His166) is essential for maintaining the catalytically competent architecture of the enzyme, whereas the neighbouring aromatic residues, particularly Trp157, predominantly mediate substrate recognition and stabilization. Experimental CRISPR/Cas9 cleavage assays further validated the computationally prioritized H147 and W157 target sites, establishing a direct link between structural bioinformatics and practical genome-editing applications. Beyond elucidating the molecular mechanism of ZmGA20ox3, this work establishes a generalizable pipeline for structure-guided genome editing in plants. By integrating AI-assisted protein structure prediction, active-site characterization, evolutionary conservation, computational mutagenesis, molecular docking, molecular dynamics simulations, binding free-energy analyses, and experimental validation, the proposed workflow enables rational prioritization of functionally important editing targets before undertaking labour-intensive wet-lab experiments. Unlike conventional sequence-based guide RNA design, this framework incorporates structural and mechanistic information to predict the functional consequences of targeted mutations. Consequently, the pipeline can be readily adapted to diverse protein-coding genes, metabolic pathways, and crop species lacking experimentally resolved protein structures, providing a predictive and cost-effective strategy for precision genome editing and accelerating next-generation crop improvement.

## Data Availability

The datasets presented in this study can be found in online repositories. The names of the repository/repositories and accession number(s) can be found in the article/**Supplementary Material**.
