# Supplementary material for: Structure-guided identification of catalytic determinants in ZmGA20ox enables rational CRISPR target selection for gibberellin engineering in maize
Source: Front Plant Sci. 2026 Sep 9;17:1948764. doi: 10.3389/fpls.2026.1948764 (PMC13597750; doi:10.3389/fpls.2026.1948764)
Supplement: Supplementary file 1 [file Table1.docx]

**Supplementary Fig 1** :Structural validation and stereochemical assessment of the predicted ZmGA20ox3 protein model.
(A) Verify3D analysis showing compatibility between the amino acid sequence and the predicted three-dimensional structural environment of ZmGA20ox3. The majority of residues exhibited acceptable environmental profile scores above the recommended threshold.
(B) Ramachandran plot generated using PROCHECK illustrating the distribution of backbone dihedral angles (φ and ψ) of amino acid residues within favoured, additionally allowed, generously allowed, and disallowed conformational regions. Most residues were localized within energetically favoured regions, indicating acceptable stereochemical geometry of the predicted structure.
(C) ERRAT quality assessment profile showing non-bonded atomic interaction statistics and overall structural reliability of the predicted ZmGA20ox3 model. Residue regions with acceptable quality scores supported the structural suitability of the model for downstream docking analyses.
(D) Residue-specific Ramachandran distribution plots representing conformational preferences of selected amino acid classes within the ZmGA20ox3 structure.

(E) AlphaFold per-residue pLDDT confidence profile, with the functionally investigated residues H147, D149, F151, W157, F163, and H166 indicated.

(F) AlphaFold predicted aligned error (PAE) heatmap showing confidence in the relative positioning of residues throughout the ZmGA20ox3 model.

**Supplementary Table 1. Hydrogen bond occupancy (propensity) of ligand 2O and GA20 with WT and mutant proteins during the 100 ns MD simulation**

| **2O** | | |
| --- | --- | --- |
| **Donor** | **Acceptor** | **Propensity (%)** |
| **H147A mutant** | ASP149 | 75.94 |
|  | ASP364 | 57.95 |
|  | CYS154 | 45.61 |
|  | SER153 | 42.86 |
| W157A  **mutant** | ASP364 | 54.02 |
|  | LYS254 | 33.13 |
|  | HIS147 | 21.59 |
|  | CYS154 | 20.84 |
| **WT** | GLU56 | 61.10 |
|  | ASP364 | 27.80 |
| **GA20** | | |
| **Donor** | **Acceptor** | **Propensity (%)** |
| **H147A mutant** | ALA147 | 6.57 |
| W157A  **mutant** | ARG58 | 45.53 |
|  | HIS166 | 31.35 |
|  | THR60 | 22.23 |
| **WT** | SER153 | 42.72 |
|  | GLN358 | 19.78 |

**A)RMSF graph for 2O**

**B)RMSF graph for GA20**

**Supplementary Figure 4.** RMSF profiles of the protein backbone during the 100 ns molecular dynamics simulation for ligand A) 2O bound to the H147A mutant, W157A mutant, and WT protein, and ligand B) GA20 bound to the H147A mutant, W157A mutant, and WT protein.

A) 2O

B) GA20

**Supplementary Figure 5.** Number of hydrogen bonds formed per frame during the 100 ns molecular dynamics simulation for ligand A) **2O** and B) **GA20** complexes with the H147A mutant, W157A mutant, and WT proteins.

**Supplementary Figure 6.** Agarose gel electrophoresis showing the in vitro SpCas9 cleavage assay for gRNAs targeting genomic regions encoding H147 (g1) and W157 (g2) of *ZmGA20ox3*. **M**, DNA ladder(100 bp); **C**, undigested PCR amplicon (670 bp); **g1 (H147)**, Cas9-mediated cleavage reaction producing the expected ~441-bp and ~229-bp products; **g2 (W157)**, Cas9-mediated cleavage reaction producing the expected ~471-bp and ~199-bp products. The cleavage pattern indicates sequence-specific SpCas9 activity directed by the respective gRNAs under the tested in vitro conditions.

**Supplementary Figure 2.** Heatmap showing the molecular docking scores (kcal mol⁻¹) of wild-type (WT) ZmGA20ox3 and alanine-substituted mutants (H147A, D149A, H166A, W157A, F163A, and F151A) against GA12, GA20, GA53, 2-oxoglutarate (2OG), and prohexadione. More negative docking scores indicate more favorable predicted ligand binding. The catalytic-site substitutions H147A, D149A, and H166A generally exhibited less favorable predicted docking scores than the WT protein, whereas substitutions at the substrate-recognition residues showed ligand-dependent effects. These results indicate that the investigated residues influence the predicted ligand-binding environment of ZmGA20ox3, although docking scores alone do not establish experimental binding affinity or catalytic activity.

| A) Ligand RMSD graph for 2-Oxoglutarate | |
| --- | --- |
|  |  |
| B) Ligand RMSD graph for GA20 | |
|  |  |

**Supplementary Figure 3**. Ligand RMSD profiles of 2O and GA20 during 100 ns molecular dynamics simulations. (A) Ligand RMSD of 2O in the WT, H147A, and W157A protein complexes. (B) Ligand RMSD of GA20 in the WT, H147A, and W157A protein complexes. RMSD values are expressed in nm and were calculated over the 100 ns MD trajectories after fitting the trajectories to the protein backbone. The profiles represent ligand positional fluctuations relative to the reference conformation throughout the simulations. H147A, histidine-to-alanine substitution at position 147; W157A, tryptophan-to-alanine substitution at position 157; WT, wild type

**Supplementary Figure 7.** Gibberellin biosynthetic pathway associated with GA20-oxidase activity and rationale for ligand selection. The diagram illustrates the two major GA20ox-associated branches of gibberellin biosynthesis. In the non-13-hydroxylation pathway, GA12 undergoes sequential oxidation through GA15 and GA24 to form GA9. In the early-13-hydroxylation pathway, GA53 is sequentially oxidized through GA44 and GA19 to form GA20, which can subsequently serve as a substrate for GA3-oxidase-mediated conversion to bioactive GA1. GA12 and GA53 were selected as representative upstream substrates of the two branches, whereas GA20 was selected as the downstream product of the GA53-derived branch and a metabolically relevant GA20ox-associated intermediate. The diagram therefore provides the biochemical rationale for selecting GA12, GA53, and GA20 for computational analysis.
